# Epidemiological Study of Parasitic Infections in BuMusa Island, Hormozgan

**DOI:** 10.18502/ijpa.v15i3.4208

**Published:** 2020

**Authors:** Hossein SOBATI

**Affiliations:** Health Research Center, Life Style Institute, Baqiyatallah University of Medical Sciences, Tehran, Iran

**Keywords:** Parasitic Infection, Human, Water, Animals, Iran

## Abstract

**Background:**

This epidemiological study aimed to investigate the prevalence of parasitic infections in BuMusa Island, Iran, in one year from 2015 to 2016.

**Methods:**

The current cross-sectional study was conducted in coordination with the health authorities of BuMusa on 732 intestinal samples and 1207 blood samples randomly collected from the island residents. Cutaneous lesions of 1207 people were clinically examined and those suspected of parasitic infections were enrolled. Also, 165 intestinal samples from domestic animals, 35 samples from water tanks, and 330 soil samples were taken to the laboratory to be investigated in terms of parasitic infections.

**Results:**

The obtained results showed 26.4% and 45.5% intestinal parasitic infections in humans and animals, respectively. The most prevalent infections in humans were *Blastocystis hominis* (8.6%), followed by *Giardia lamblia* (8.2%), and *Entamoeba coli* (6.8%); and the least prevalent infection was *Enterobius vermicularis* (<0.2%). Malaria agents and *Leishmania* were not observed in blood samples. Investigation of animal feces showed that the highest parasitic infection was *Eimeria arloingi* (16.4%), while the lowest prevalence belonged to *Monizia expansa* (0.6%). *Hymenolepis nana* eggs and *Cyclops* were detected in one sub-source of water tanks. *Rhabditis* larva, a free-living nematode, was observed in a soil sample.

**Conclusion:**

The prevalence of parasitic infections in BuMusa Island was relatively low probably due to its hot and dry climate.

## Introduction

Parasitic diseases are one of the most important health problems in the world, especially in developing countries. Parasites heavily endanger human health and cause various degrees of anemia, and mental and physical retardation, particularly in children. Therefore, controlling their spread and preventing their incidence is critically demanded (1).

Iran is one of the countries with relatively high prevalence of parasitic diseases especially in certain geographical areas (2). The wide difference between parasitic infection rates in different areas of Iran originates from the large variation in ecological, social, economic, and cultural characteristics of different regions of this country (3). The most parasitic infections are observed in the southern regions of Iran similar to other tropical and subtropical countries across the world that are challenging with leishmaniasis and malaria as significant global health issues. Despite large investments and efforts for curbing and controlling leishmaniasis and malaria, extensive parts of Iran are still involved with these serious health problems (4). The climate, weather conditions, vegetation of these regions, and the method of feeding animals are considered as effective factors that contribute to the distribution of parasitic infections, including leishmaniasis and malaria, in the south of Iran (5).

Studies have shown that soil and water, as the main reservoir for the cysts and parasite eggs, play a particularly important role in spreading the parasitic diseases. Also, infected animals such as dogs and cats excrete protozoan cysts and helminth eggs in their feces and help the parasitic agents spread across the soil and water (6).

Given the importance of parasitic diseases in tropical areas, investigation of the parasitic prevalence of a particular area, and how it is transmitted can be effective in identifying risk factors and infection control. In this regard, BuMusa Island at the south of Iran (Persian Gulf) was investigated in terms of parasitic incidence in a sample population of either human, animal, water or soil of the region. It should be noted that BuMusa County, in the Hormozgan Province, includes islets BuMusa, Greater and Lesser Tunb, Greater and Lesser Faror, and Siri. BuMusa is the Southernmost island with an area of 70.5 square kilometers and has a population of >7000 persons as 716 families. Having a tropical climate (hot and humid), the island is warmer than other Iranian islands in the Persian Gulf due to its proximity to the equator. Lack of enough water and adequate agricultural land has caused BuMusa Island to have a tropical flora with poor vegetation.

To the best of the authors knowledge, the prevalence of parasitic infections have not been studied in the BuMusa Island, therefore, the present study is the first report that has gathered relevant information in regards to the distribution of parasitic infections in this region. The current study aimed to determine the status of parasitic fauna with special reference to the pathogenic parasites in human and animal populations, soil, and drinking water. The findings of the present study are valuable for implementing the health plans for restraining the parasitic diseases in the BuMusa Island.

## Materials and Methods

The sample size required for each source (human, animal, soil, and water) was determined by an initial pilot study with a precision of 0.02 and a confidence level of 98%. The current study was a cross-sectional assessment; the city was divided into five Northern, Southern, Eastern, Western, and Central regions and sampling was performed from 2015 to 2016.

Ethics committee of Baqiyatallah University of Medical Sciences approved the study. In addition, a written informed consent was obtained from all the participants before blood and feces sampling.

### Sampling from human and animal feces

A total of 732 people living in the five determined regions with at least five years of residence in BuMusa were randomly selected. The stool samples were collected three times in labeled sampling containers and their individual characteristics were recorded in a questionnaire. Also, a total of 165 stool samples were randomly collected from domesticated animals including goats, sheep, camels, cows, and cats from each division. Then, samples were prepared in plastic containers tagged with the animal type. Some of the human and animal samples were separately transferred into screw-cap tubes with 10% formalin or polyvinyl alcohol (PVA) and sent to the laboratory. Direct, formalin-ether, flotation, and temporary Lugol’s iodine staining methods were applied to study the stool samples (7).

### Blood and tissue samples from human

A total of 1207 people were randomly selected from the five regions of BuMusa and their characteristics, including their infection history to malaria and leishmaniasis, were recorded in a questionnaire. The presence of *Plasmodium* (malaria agent) and *Leishmania* parasites in samples was investigated by blood sampling and clinical examination, respectively. Thin and thick extensions from all 1207 blood samples plus 25 wound samples with suspected leishmaniasis smears were prepared and stained with Giemsa (7). Suspected leishmaniasis samples were cultured in a biphasic NNN (Novy-MacNeal-Nicolle) medium. The culture media were incubated at 18–25°C for 3–4 weeks. Every four days, a sample was taken from the liquid portion. Blood, culture, and wound samples were then examined by microscopy.

### Soil sampling

To investigate the presence of parasitic agents (i.e., protozoan cysts, helminth eggs, or larvae) in the soil, 330 samples were collected from five regions of BuMusa within a year (165 samples per six months). Soil samples were randomly taken from the surface to a depth of 15 cm by sterile spatula, then packaged in sterile containers and transferred to the laboratory where 30 g of each soil sample was weighed and transferred to a medium beaker and mixed with 100 mL of physiologic serum to obtain a homogenous suspension. The suspensions were passed through a filter and then centrifuged at 2000 rpm for three to five minutes. The supernatants were discarded and sediments were tested by direct smear, formalin ether, flotation, and Lugol iodine staining (7).

### Sampling of water

To investigate the parasitic contamination of major and minor tanks of drinking water, 100 water samples were collected from available sources in the five above-mentioned regions at a rate of about 2000 liters. Water samples were prepared according to the World Health Organization (WHO) instruction with a filter membrane method using the vacuum pump. Then samples were passed through 0.8 μm pore size cellulose acetate filter (Germany). The deposits remained on the filter were washed with sterile water and collected and deposit-containing solutions were divided into two portions. One portion was transferred to sterile screw-cap containers and the other one to containers containing 10% formalin, both sent to the laboratory for further experiments. All samples were evaluated with the direct method, formalin ether, and Lugol iodine staining. If coccidian shapes were observed or the case was suspected, Sheather’s sugar flotation technique and modified Ziehl-Neelsen staining method were further performed (7).

### Statistical analysis

Data were analyzed with SPSS version 19 using the Chi-square test. A *P*-value < 0.05 was considered as the level of significance.

## Results

### The results of intestinal samples from humans

Results showed that among the studied human intestinal samples, 26 (~11.6%) were contaminated mostly aged in the range of 20 to 24 years old. The highest and lowest intestinal parasitic infection related to *Blastocystis hominis* (8.6%) and *Enterobius vermicularis* (0.14%), respectively (Table 1).

**Table 1:** The prevalence of intestinal infections in BuMusa residents based on age

***Age*** ***Identified parasite***	***20 > No, (%)***	***20 – 24 No, (%)***	***25 – 29 No, (%)***	***30 – 34 No, (%)***	***35 – 39 No, (%)***	***40 ≤ No, (%)***	***Total Number (%)***
*Giardia lamblia*	16 (2.19)	29 (3.96)	6 (0.82)	4 (0.55)	4 (0.55)	1 (0.14)	60 (8.20)
*Entamoeba coli*	13 (1.78)	25 (3.42)	3 (0.41)	4 (0.55)	2 (0.27)	3 (0.41)	50 (6.83)
*Entamoeba histolytica/dispar*	0	1 (0.14)	1 (0.14)	0	0	0	2 (0.27)
*Blastocystis hominis*	18 (2.46)	24 (3.28)	4 (0.55)	9 (1.23)	5 (0.68)	3 (0.41)	63 (8.60)
*Endolimax nana*	0	1 (0.14)	1 (0.14)	0	0	0	2 (0.27)
*Hymenolepis nana*	8 (1.09)	4 (0.55)	1 (0.14)	0	2 (0.27)	0	15 (2.05)
*Enterobius vermicularis*	0	1 (0.14)	0	0	0	0	1 (0.14)
Infected	55 (7.51)	85 (11.61)	16 (2.19)	17 (2.32)	13 (1.78)	7 (0.96)	193 (26.37)
Not-infected	120	250	43	46	40	40	539 (73.63)
Total	175	335	59	63	53	47	732

However, no statistically significant relationship was observed between the infection rate and age. However, there was a significant difference between infection risks in various age groups (*P* < 0.05). There was also a significant relationship between the risks of infection in people with different education levels (*P* < 0.05). The highest rate of infection was observed among illiterate people (Table 2).

**Table 2:** The prevalence of parasitic contamination in BuMusa residents based on education level

***Groups*** ***Education level***	***Infected to parasite No, (%)***	***Not-infected No, (%)***	***Total No.***
Uneducated	23 (62.16)	14 (37.84)	37
Elementary level	62 (28.70)	154 (71.30)	216
High level	108 (22.55)	371 (77.45)	479
Total	193 (26.37)	539 (73.63)	732

Also, the infection rate in males was about 26.1% and in females 27.7%. The highest and lowest infection rate in males belonged to *B. hominis* (8.7%) and *E. vermicularis* (0.16%), respectively.

In females, the contamination was mostly related to *G. lamblia* (9.8%) and *Hymenolepis nana* (1.8%) while *Entamoeba histolytica/dispar* had the lowest rate of infection among females. *E. histolytica/dispar*, *E. vermicularis* and *Endolimax nana* were not observed in the studied population (Table 3).

**Table 3:** The prevalence of parasitic contamination in BuMusa residents based on Sex

***Sex*** ***Identified parasite***	***Male No, (%)***	***Female No,(%)***	***No, (%)***
*Giardia lamblia*	49 (7.90)	11 (9.82)	60 (8.50)
*Entamoeba coli*	43 (6.93)	9 (8.03)	50 (7.08)
*Entamoeba histolytica/dispar*	2 (0.32)	0	2 (0.28)
*Blastocystis hominis*	54 (8.71)	9 (8.03)	63 (8.92)
*Endolimax nana*	2 (0.32)	0	2 (0.28)
*Hymenolepis nana*	13 (2.10)	2 (1.79)	15 (2.12)
*Enterobius vermicularis*	1 (0.16)	0	1 (0.14)
Infected	162 (26.12)	31 (27.68)	193 (23.65)
Not-infected	458	81	539 (76.35)
Total	620	112	732 (100)

The infection rate of intestinal parasites was 26.5% for civilian residents and 26.3% for military residents. The highest contamination rate in military residents associated with *G. lamblia* with 9.4% and the lowest to *E. vermicularis* by 0.2% prevalence rates (Table 4).

**Table 4:** Comparison of the prevalence of parasitic contamination in between military or general patients in BuMusa Island

***Occupation*** ***Identified parasite***	***Military residents No, (%)***	***Civilian residents No, (%)***
*Giardia lamblia*	49 (9.40)	11 (5.21)
*Entamoeba coli*	36 (6.91)	14 (6.64)
*Entamoeba histolytica/dispar*	2 (0.38)	0
*Blastocystis hominis*	38 (7.29)	25 (11.85)
*Endolimax nana*	2 (0.38)	0
*Hymenolepis nana*	9 (1.73)	6 (2.84)
*Enterobius vermicularis*	1 (0.19)	0
Infected	137 (26.30)	56 (26.54)
Not-infected	384	155
Total	521	211

In general, the highest infection rate belonged to *B. hominis* by 11.9% and the lowest to *H. nana* (~2.8%). The infection distribution of parasites displayed no significant difference between the two sub-populations. However, the statistical difference of the contamination type between civilian and military residents was significant (*P* < 0.05).

Results also represented the contamination ratio in the elementary, secondary, and high school students to be 34.2% in total with a higher denotative rate in the elementary school students (Table 5). Infection with the helminth parasite *H. nana* was observed only in elementary school students. The total prevalence of parasitic helminths and protozoa was 2.2% and 24.2%, respectively, while this ratio was around 5.8% and 28.3% for students. Thus, results showed the superiority of the protozoal infections compared to helminth agents based on their statistically significant difference (*P* < 0.05).

**Table 5:** The prevalence of parasitic infection in school students in BuMusa Island

***Grade*** ***Identified parasite***	***Primary school No, (%)***	***Guidance school No, (%)***	***High school No, (%)***	***No, (%)***
*Giardia lamblia*	4 (5.48)	1 (0.83)	5 (4.16)	10 (8.33)
*Entamoeba coli*	3 (4.11)	4 (3.33)	2 (1.66)	9 (7.50)
*Blastocystis hominis*	9 (12.33)	4 (3.33)	2 (1.66)	15 (12.50)
*Hymenolepis nana*	7 (5.83)	0	0	7 (5.83)
Infected	23 (19.16)	9 (7.50)	9 (7.50)	41 (34.17)
Not-infected	50	16	13	79 (65.83)
Total	73	25	22	120 (100)

### Parasitic prevalence in human blood and tissue

Malaria parasite and cutaneous leishmaniasis were not observed in the 1207 cases through blood smears and physical examinations. Among 25 cases suspected of cutaneous leishmaniasis, no infection was detected through NNN culture. However, 51 subjects had a history of leishmaniasis and 50 subjects had a history of malaria. The etiology of their infection was chiefly recognized to be a trip to Hormozgan, Sistan and Baluchistan, and Khuzestan provinces.

### Parasitic prevalence in fecal samples of domestic animals

An intestinal parasite prevalence of 45.5% was recorded in 165 domestic animal fecal samples from five different regions of BuMusa. The results showed the highest and lowest infection prevalence in goat and sheep associated with *Entamoeba bovis* (37.9%) and *Moniezia expansa* (1.72%), respectively (Table 6). In camels, the highest and lowest infection rates belonged to *Eimeria arloingi* (22.5%) followed by *Balantidium coli* (2.8%). Other parasites including *Trichuris ovis*, *M. expansa*, *Capillaria obsignata*, *Strongyloides* spp., *Giardia* spp., and *E. bovis* were not observed. In cats, only *Strongyloides* spp. with the rate of 6.7% was detected, while in birds, *C. obsignata* and *Strongyloides* spp. parasites were equally observed (14.3%). The incidence of parasitic helminths and protozoan in domestic animals were overall 10.3% and 35.2%, respectively, and the parasitic types were significantly different among various types of animals (*P* < 0.05).

**Table 6:** The prevalence of parasitic infection in domestic animals of BuMusa Island

***Animal*** ***Identified parasite***	***Goat/Sheep No, (%)***	***Camel No, (%)***	***Birds No, (%)***	***Cat No, (%)***	***No, (%)***
*Trichostrongylus spp.*	2(3.44)	6(8.45)	0	0	8(4.84)
*Trichuris ovis*	2(3.44)	0	0	0	2(1.21)
*Moniezia expansa*	1(1.72)	0	0	0	1(0.61)
*Capillaria obsignata*	0	0	3(14.29)	0	3(1.82)
*Strongyloides spp.*	0	0	3(14.29)	1(6.66)	4(2.42)
*Balantidium coli*	0	2(2.82)	0	0	2(1.21)
*Eimeria arloingi*	11(18.96)	16(22.53)	0	0	27(16.36)
*Giardia spp.*	6(10.34)	0	0	0	6(3.64)
*Entamoeba bovis*	22(37.93)	0	0	0	22(13.34)
Infected	44(75.86)	24(33.80)	6(28.58)	1(6.66)	75(45.45)
Not-infected	14	47	15	14	90(54.55)
Total	58	71	21	15	165(100)

### Parasitic prevalence in soil samples

During the examination of 165 soil samples in the first six months, no parasitic agents such as cysts, eggs, and larvae of pathogenic parasites were observed. However, in 30 out of 165 soil samples collected in the second six months, the rhabditoid larvae of *Rhabditidea* (a free-living nematode) were observed mostly around shrubs and areas with more moisture.

### Parasitic prevalence in water samples

Investigation of 100 samples from different drinking water sources showed that *H. nana* eggs and *Cyclops* existed in one of the secondary sources. In other samples prepared from major and minor tanks, no parasitic infection was observed.

## Discussion

The importance of parasitic infections and their challenge for the developing countries necessitates basic studies on their general epidemiology, especially using intestinal samples. Such studies are required for arranging effective health strategies to confine the transmission of common infectious diseases. For the same purpose, the current research was performed to evaluate the prevalence of parasitic infections in BuMusa Island.

According to the obtained findings, 26.4% of BuMusa residents were infected with intestinal parasites. The most prevalent protozoan infections were *B. hominis*, *G. lamblia*, *E. coli*, *E. histolytica/dispar*, and *E. nana*, respectively, which was more or less similar to the findings from the other regions of the country (8, 9). For instance, the prevalence of intestinal parasites is considered to be 28% in Jiroft, Kerman Province, Iran. Actually, a total prevalence rate of 27.4% for protozoan parasites has been reported comprising a rate of 13.7% and 7.8% for *B. hominis* and *G. lamblia*, respectively, as the most prevalent parasites (10). Another epidemiological study showed that the general contamination level in school students in Tehran was less than that of Southern regions of the country, probably due to accessibility to healthier tap water and better economic condition (11). However, based on their study, again *B. hominis* and *G. lamblia* were the most prevalent parasites in Tehran. According to the aforementioned reports, the results of the studies on the contamination rate with protozoans in Southern regions of Iran nearly corresponding to our findings. The geographical and cultural congruities, including similar consumption pattern of healthy comestibles, are addressed as underling reasons for this consistency in dominant species prevalent in different regions of the country.

On the other hand, any tiny change in the geographical parameters and abiding by the sanitary disciplines alter the dominant protozoa species or cause relative fluctuation in their general contamination rate. Also, the variations in social characteristics and life styles in different cities are presumed to be the underlying reason of the statistically significance different infection rates reported by the current and other similar studies. However, accessibility to the health water seems to be a chief cofactor of protozoan infection distribution which need a separate study to be independently investigated.

Among all parasitic infections, malaria and leishmaniasis are of special importance due to their serious consequences (12). In Iran, the compromised sub-population exposed to malaria constitutes about 4% of the overall population, mostly living in Southern provinces such as Sistan and Baluchistan, Kerman, and Hormozgan. Another study examined the arthropod fauna of the islands BuMusa, Greater Tunb, and Lesser Tunb within a year and reported the presence of *Culex* mosquitoes, flies, and ticks, however, their observations stated no *Anopheles* and *Phlebotomus* mosquitoes as vectors of malaria and leishmaniasis, respectively (13). In a survey on malaria in Jask Harbor in South and Southeastern Iran from 2006 to 2010, species of *P. vivax* and *P. falciparum* and two main carriers - *Anopheles stephensi* and *A. culicifacies* were identified (14). Contrariwise, the current study showed no contamination blood and tissue parasites such as malaria and Leishmania agents in blood samples, clinical examinations, and cultures of suspected cutaneous lesion samples of leishmaniasis. These results along with findings on lack of *Anopheles* and *Phlebotomus* mosquitoes can be interpreted as the ecological and environmental conditions and parameters in BuMusa are not favorable for the occurrence and transmission of malaria and Leishmania. Investigating the intestinal parasite types in animals as the main hosts and reservoirs is vital for determining the human-animal common intestinal parasite. Hence, such studies are strongly suggested to be performed in other cities across the country to be exploited for health codifications. The current study, for the first time, has observed the reservoir distribution of common parasites in BuMusa. Accordingly, the overall intestinal parasitic infection in animals was 45.5%. *E. arlonji* and *E. bovis* were the most prevalent infectious protozoa and *Trichostrongylus* spp. was the most prevalent helminth.

Furthermore, few studies have been conducted in Iran that have surveyed the extent of soil contamination with parasites such as *Toxocara*. In 2016, the soil of 15 parks in Arak, Markazi Province, Iran was studied in order to look for *Toxocara*, and indicated *Toxocara* eggs in the soil of four parks (26.6%) (15). But in the current study, no parasitic species except free-living rhabditiform larvae were identified in soil samples, which seems to be due to lack of suitable environmental conditions. Assumably, the high temperature inhibits the stability and survival of parasitic eggs/cysts in the soil of BuMusa. The low intestinal parasitic infection transmitted by the soil in the intestinal samples of BuMusa confirms the aforementioned assumption. On the other hand, the prevalence of some parasitic diseases associates with water quality directly or indirectly. Generally, the incidence and prevalence of water-borne protozoans are usually more than those of helminth infections (16). In developing countries, *Giardia* and *Cryptosporidium* are the main causes of water-borne diseases. These two protozoa exist in a large number of native animals and the density of their infectious cysts in the culture medium is enough for water pollution. Additionally, the cysts releasing in water sources are resistant to disinfectants and drugs (17). Based on studies conducted in both the United States and Iran, drinking water is reported to be the main source of *Giardia* infection causing epidemic Giardiasis (16, 18). In Iran, the intestinal parasites of the military forces in Bandar Abbas and the islands of Greater Tunb and Lesser Tunb have been studied by sampling bottled water, water tanks, and the tap water of the barracks. Accordingly, a contamination rate of 29.8% mostly related to *E. histolytica*, *Giardia*, *Ascaris*, and *H. nana* has been revealed (19). In this study, *Giardia* transmission is discussed to be mostly acquired from the drinking water rather than food and vegetables. The estimated contamination level in the current study almost corresponded to that of the results of other studies report probably because of the similar characteristics of the two regions and their specifications (16, 18, 19). However, there is a marginal difference between these two set of findings likely due to that their study only covered the military forces affected by several displacements and poor sanitation principles. On the other hand, the sanitary conditions during water transport in BuMusa could be another reason for its very low parasitic contamination of water. Therefore, paying more attention to hygiene principles in water transport and using fiberglass tanks with proper coverage, protection, etc. in water preparation and treatment procedures seem to be considerably effective in reducing the rate of contamination with parasitic and protozoan agents.

## Conclusion

Generally, findings of the current study showed that parasitic infections, especially intestinal parasites, are still a major public health challenge in Iran. It seems that observations such as providing and consuming safe and healthy drinking water, healthy food and vegetables, personal and public health education, improving environmental sanitary conditions, public participation in health programs, and supporting them in BuMusa should be specifically considered by the health officials. These points can be the key factors of success in preventing the spread of parasitic infections.
